# Supplementary material for: Primate TRIM34 is a broadly-acting, TRIM5-dependent lentiviral restriction factor
Source: Retrovirology. 2023 Aug 22;20:15. doi: 10.1186/s12977-023-00629-4 (PMC10464172; doi:10.1186/s12977-023-00629-4)

Supplemental Data Chromatogram 1

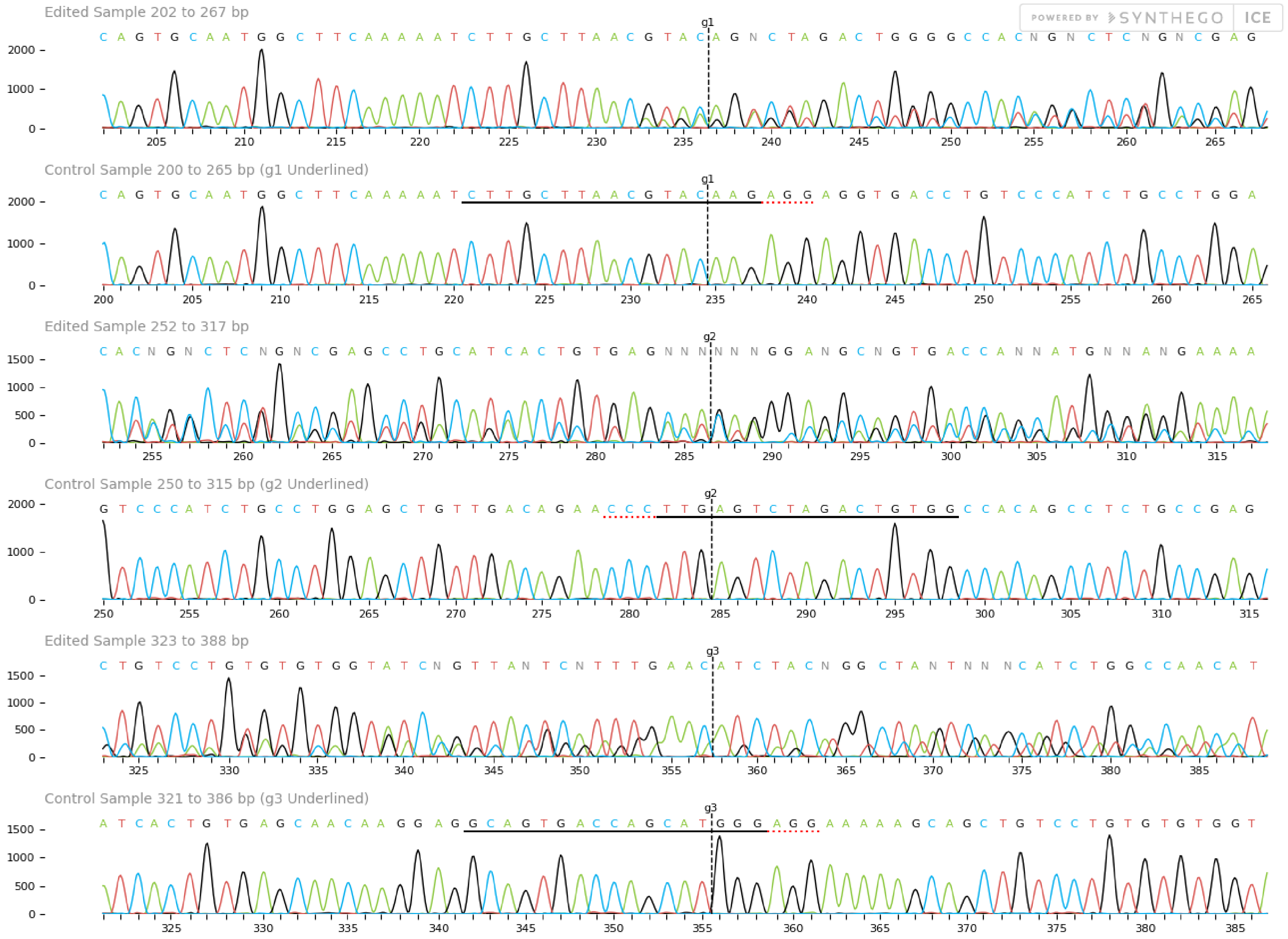

Supplemental Data Chromatogram 2

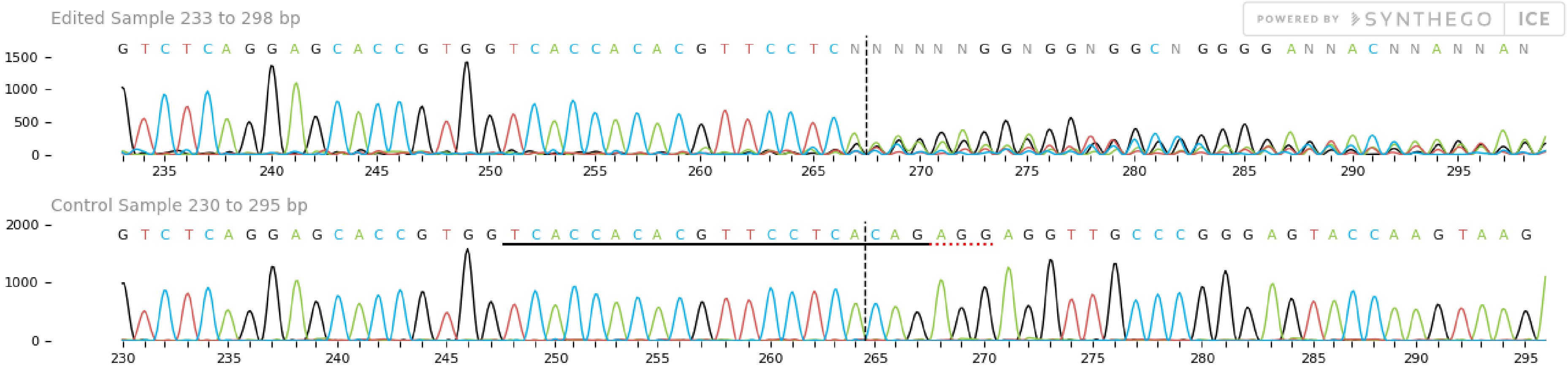

Supplemental Data Chromatogram 3

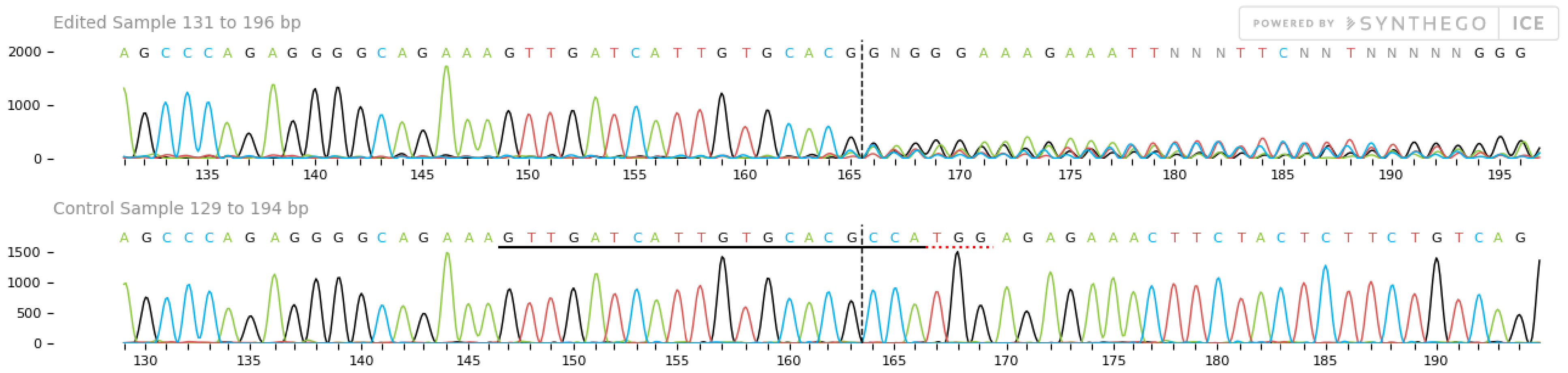

Supplemental Data Chromatogram 4

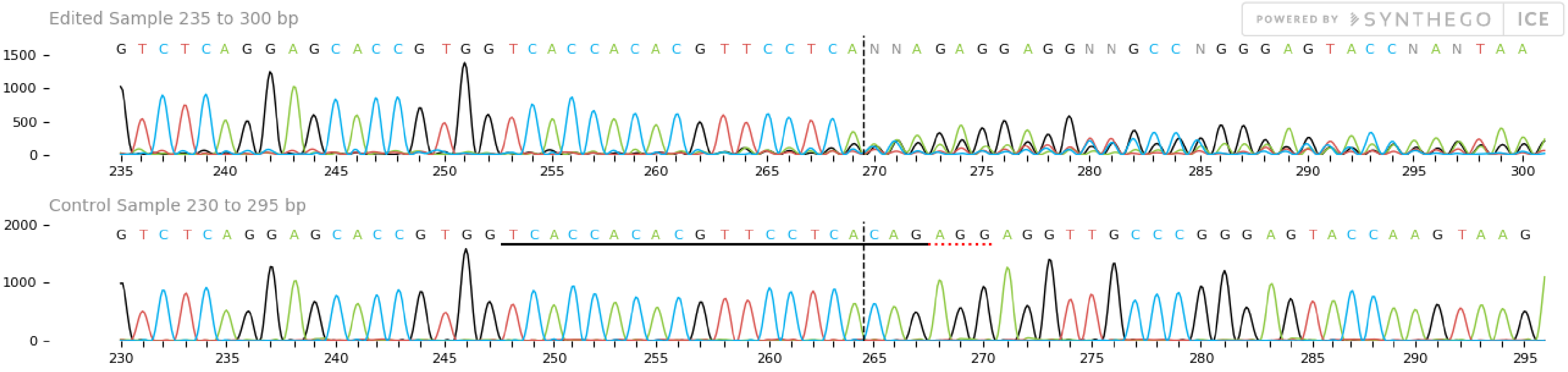

Supplemental Data Chromatogram 5

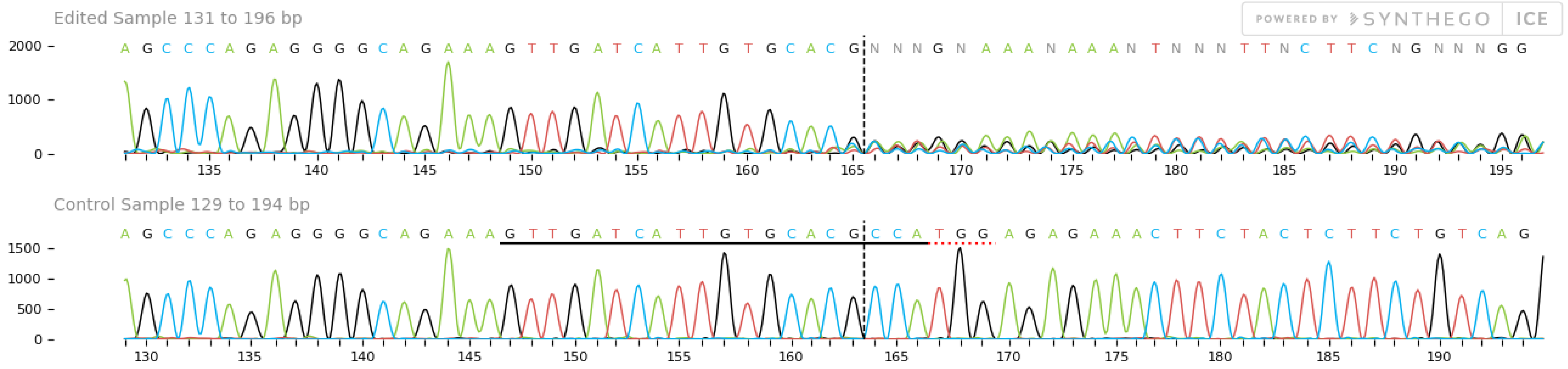

Supplemental Data Chromatogram 6

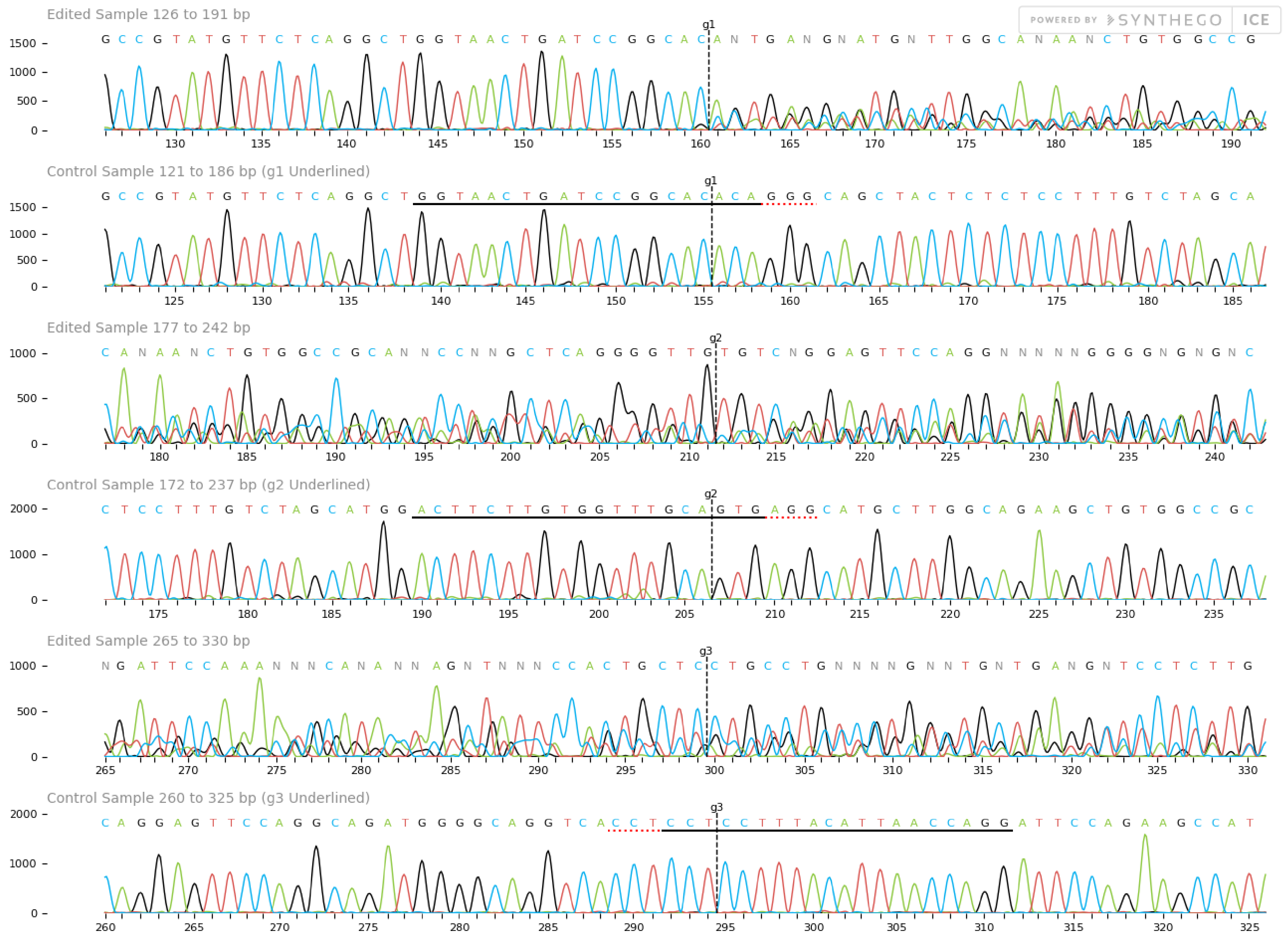

Supplemental Data Chromatogram 7

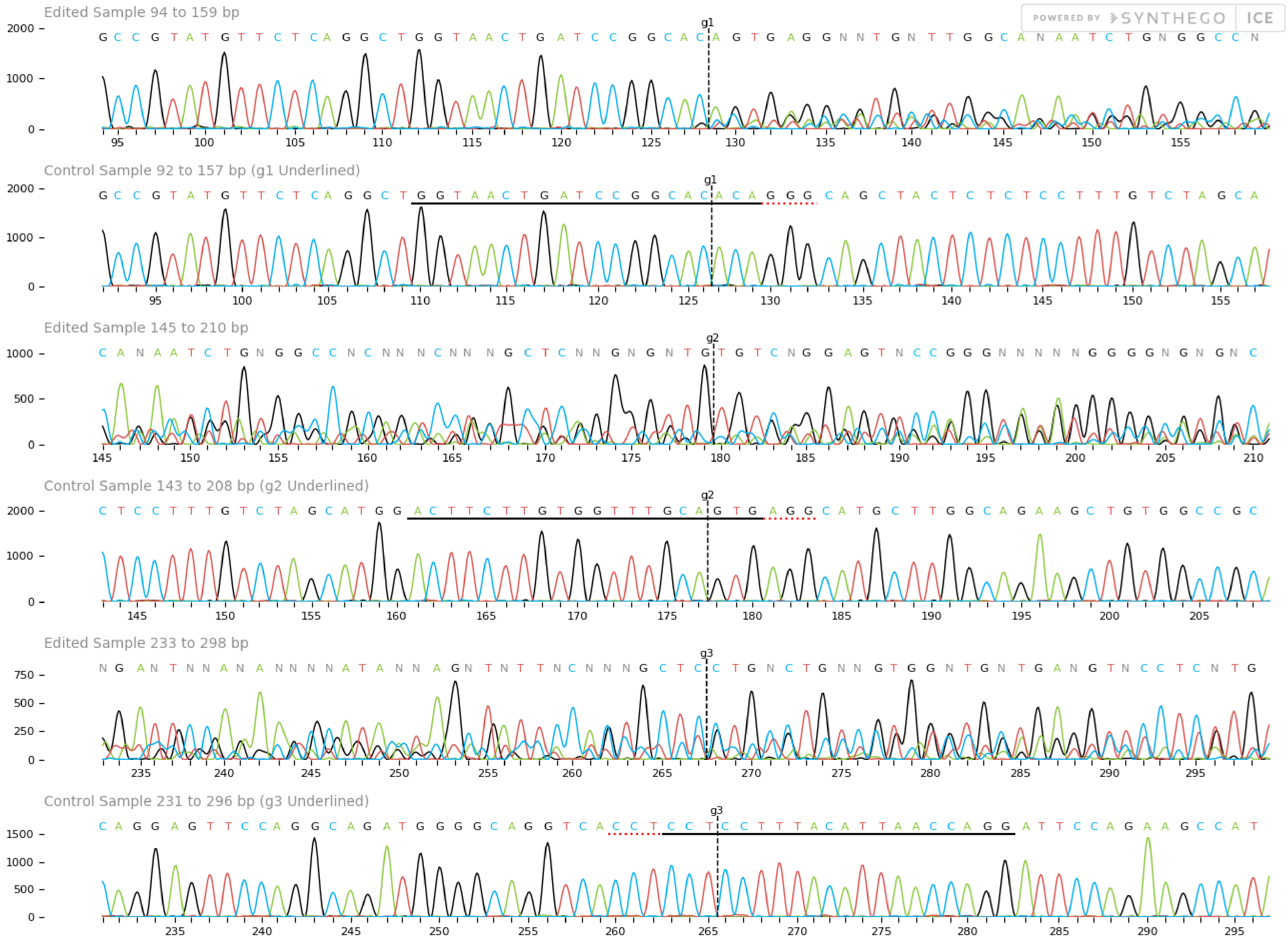

Supplemental Data Chromatogram 8

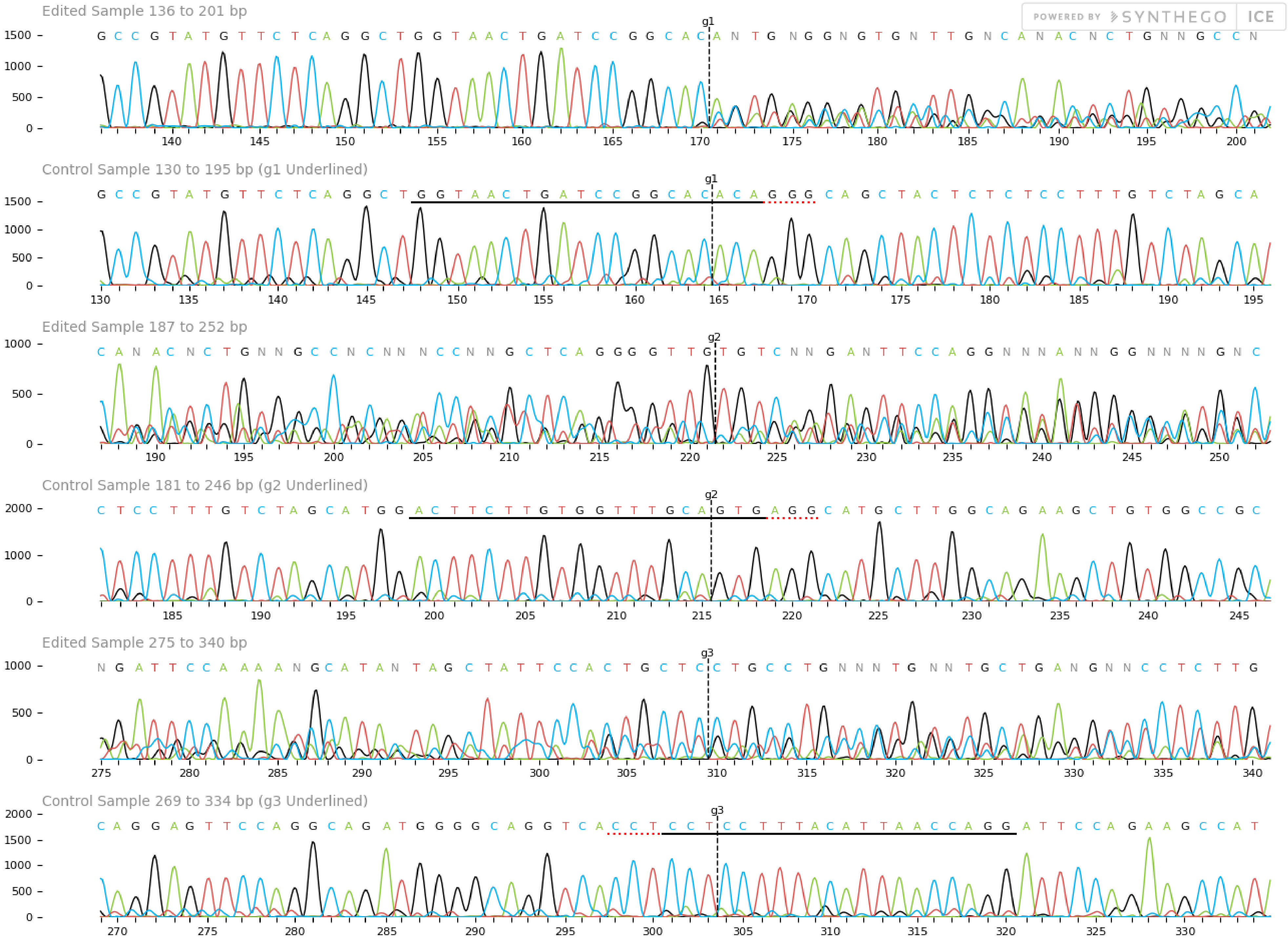

Supplemental Data Chromatogram 9

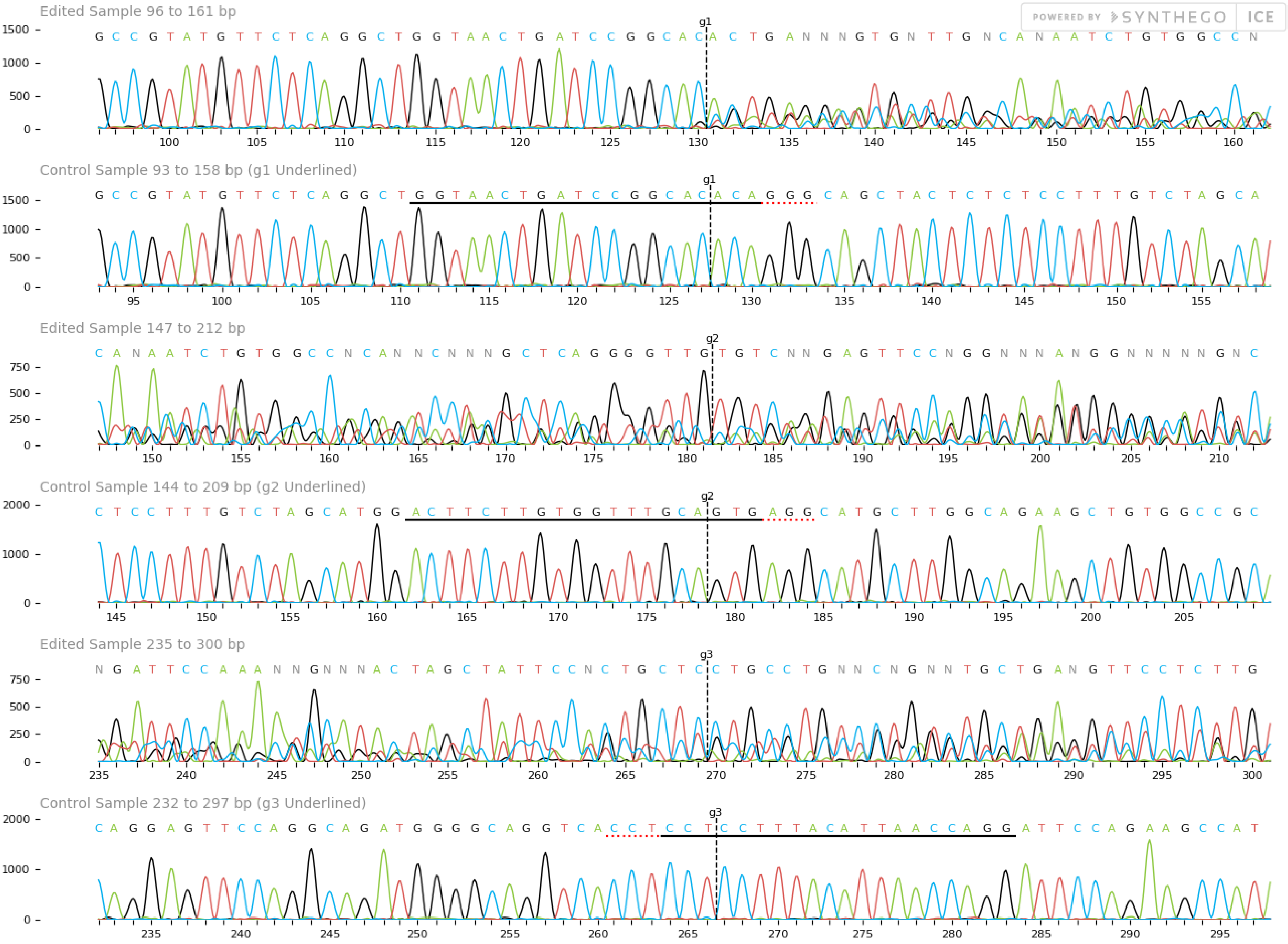

Supplemental Data Chromatogram 10

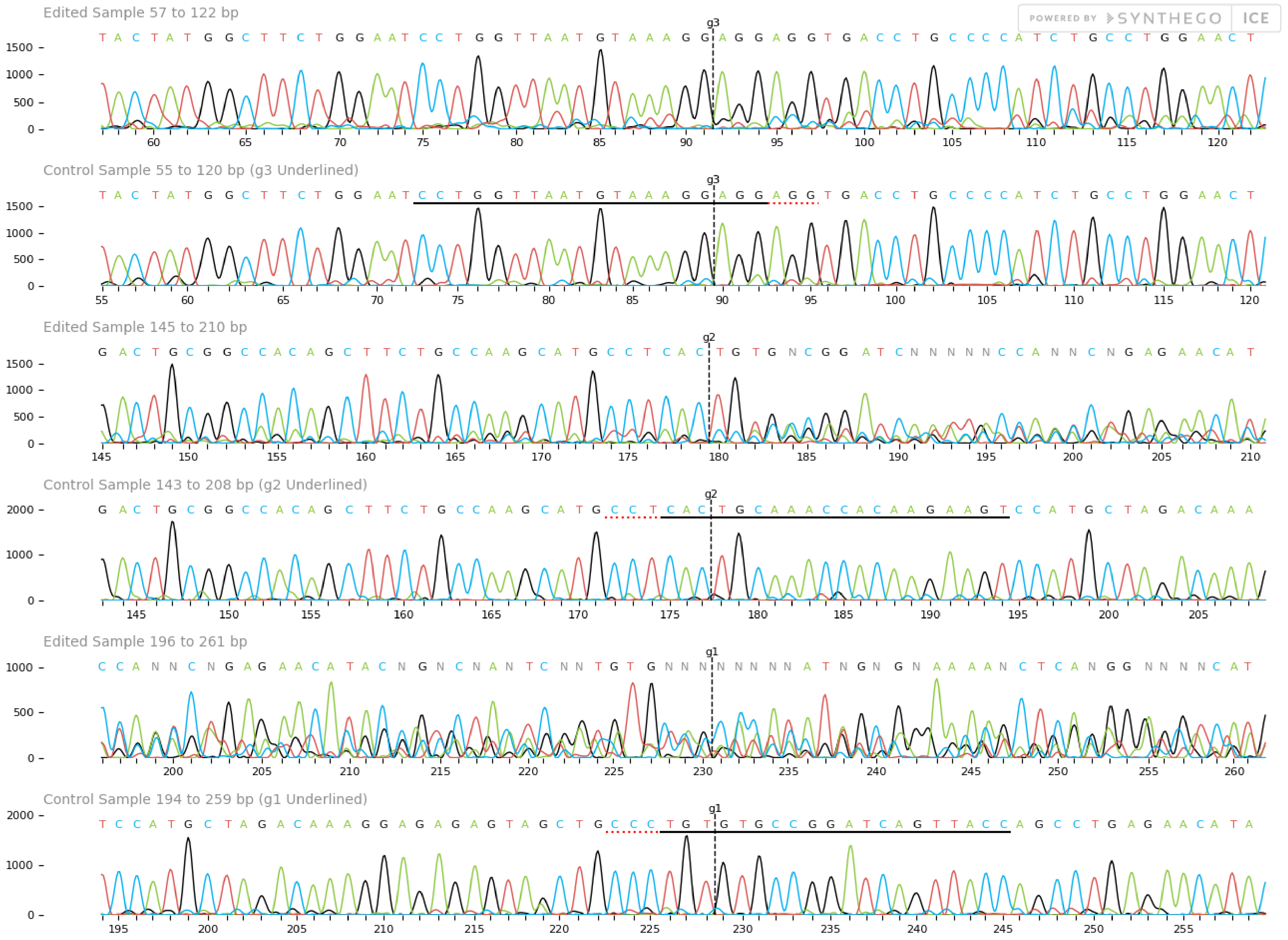

Supplement: Supplementary file 1 — Additional file 1. ICE chromatograms. [file 12977_2023_629_MOESM1_ESM.pdf]
