# Supplementary material for: Primate TRIM34 is a broadly-acting, TRIM5-dependent lentiviral restriction factor
Source: Retrovirology. 2023 Aug 22;20:15. doi: 10.1186/s12977-023-00629-4 (PMC10464172; doi:10.1186/s12977-023-00629-4)

Supplemental data blot 1a

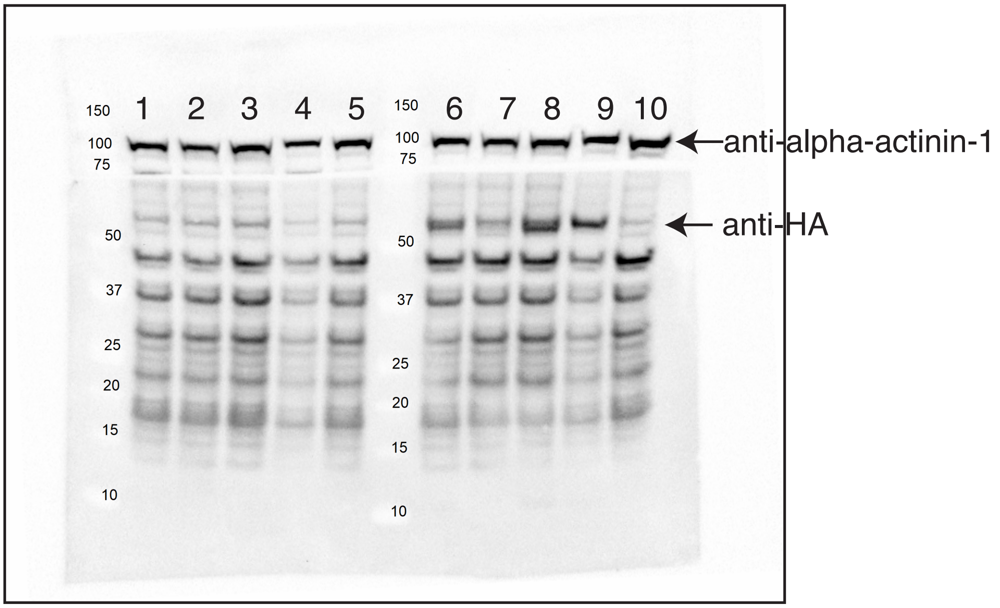

Supplemental data blot 2c

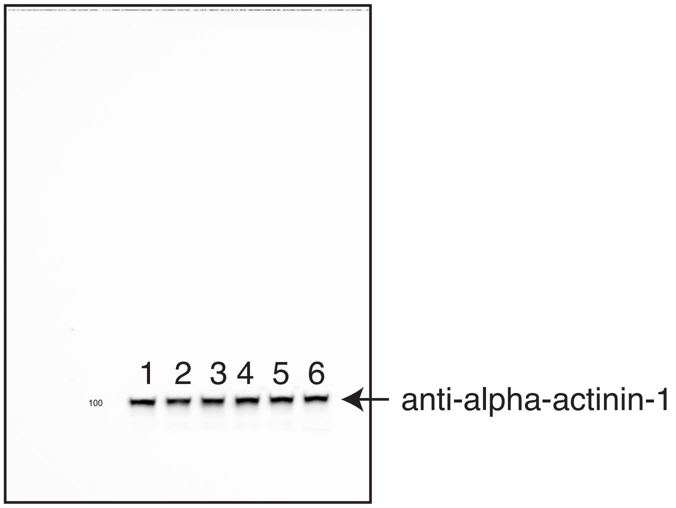

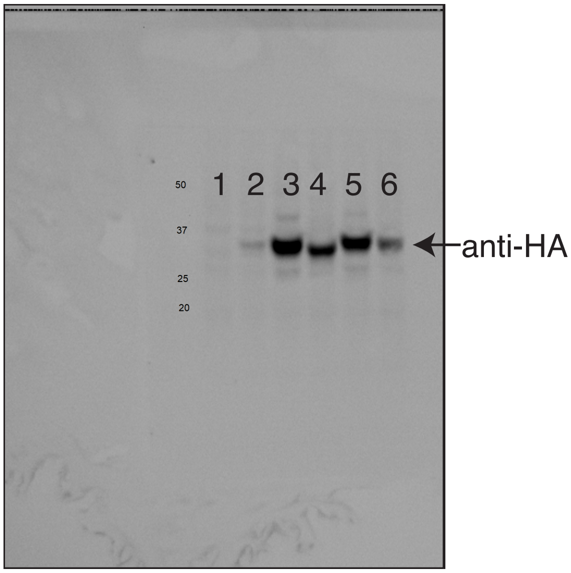

Supplemental data blot 3a

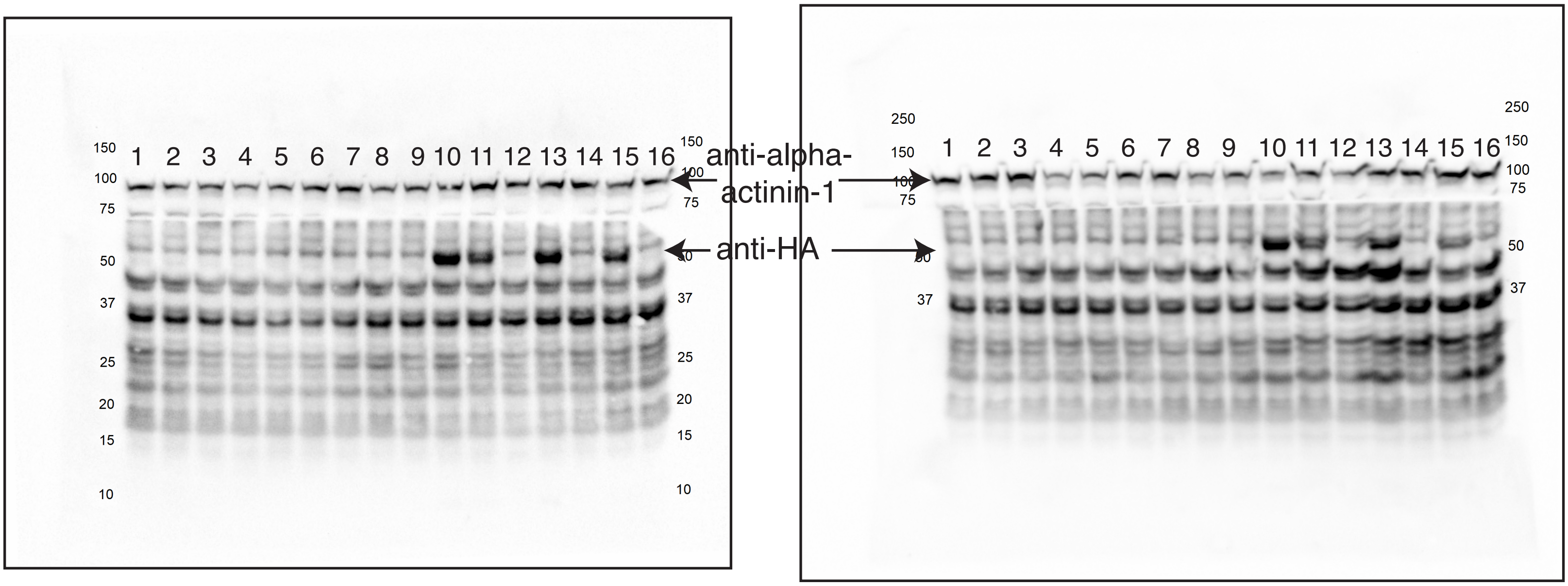

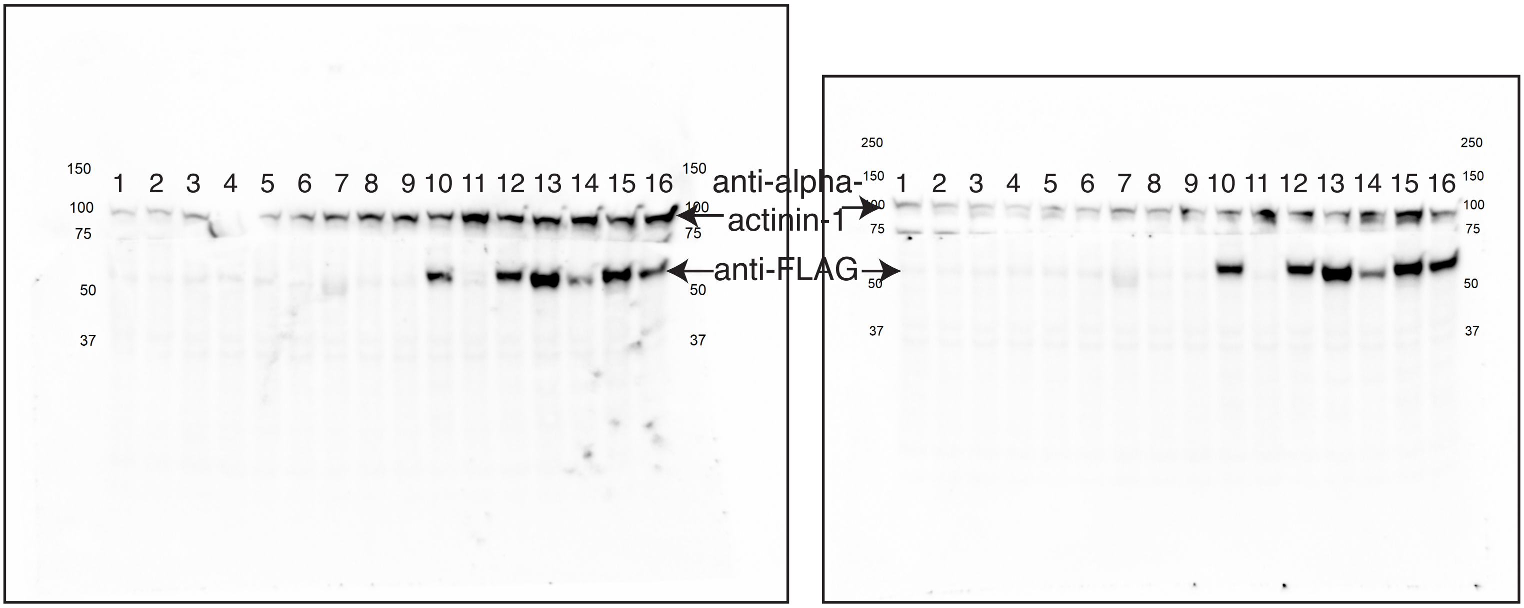

Supplemental data blot 4a

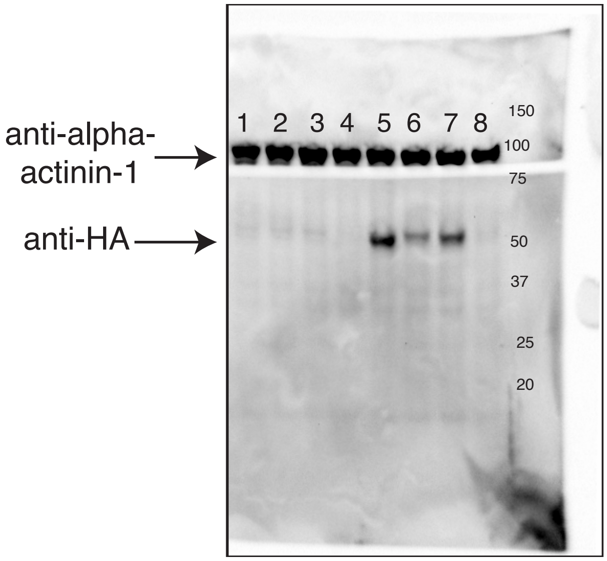

Supplement: Supplementary file 3 — Additional file 3. Full Western blot membranes. [file 12977_2023_629_MOESM3_ESM.pdf]
